# Association between caring for grandchildren based on living arrangements and cognitive function among Chinese middle-aged and older adults: The mediating roles of social activities and depressive symptoms

**DOI:** 10.3389/fpubh.2023.1105066

**Published:** 2023-02-14

**Authors:** Xuebei Hou, Yuan Xiong, Guiyuan Qiao, Jing Zhou

**Affiliations:** ^1^School of Nursing, Hubei University of Chinese Medicine, Wuhan, China; ^2^Department of Tuina and Rehabilitation Medicine, Hubei Provincial Hospital of Traditional Chinese Medicine, Wuhan, China; ^3^Department of Tuina and Rehabilitation Medicine, Affiliated Hospital of Hubei University of Chinese Medicine, Wuhan, China; ^4^Department of Tuina and Rehabilitation Medicine, Hubei Institute of Traditional Chinese Medicine, Wuhan, China; ^5^First Clinical Medical College, Hubei University of Chinese Medicine, Wuhan, China

**Keywords:** cognitive function, social activities, depressive symptoms, living arrangements, caring for grandchildren

## Abstract

**Introduction:**

In the context of an aging population and age-related conditions increasing, the increasing number of middle-aged and older adults are involved in grandchildren care. This study aimed to 1) explore the association between caring for grandchildren based on living arrangements and cognitive function among Chinese middle-aged and older adults; and 2) investigate the mediating roles of social activities and depressive symptoms in the aforementioned association.

**Methods:**

This study selected 5490 Chinese people (≥45 years old) from the 2018 China Health and Retirement Longitudinal Study (CHARLS). Participants answered questions related to socio-demographics, the Mini-mental State Examination, the intensity of grandchildren care, the Center for Epidemiological Studies Depression Scale, and social activity.

**Results:**

The results showed that caring for grandchildren and cohabiting with a spouse was positively associated with cognitive function among Chinese middle-aged and older adults (B = 0.829, *p* < 0.001). Furthermore, there was a positive association between providing intensive or no-intensive grandchildren care and cognitive function. In contrast, caring for grandchildren but not cohabiting with a spouse was negatively associated with cognitive function (B = −0.545, *p* < 0.05). Moreover, directly and indirectly, caring for grandchildren was significantly associated with cognitive function among Chinese middle-aged and older adults, as mediated by social activities and depressive symptoms.

**Discussion:**

The findings emphasize that living arrangements, social engagement, and psychological health could be considered when encouraging grandparent care as formal care.

## 1. Background

Population aging is of significant importance, which leads to a substantial increase in age-related conditions, with far-reaching implications for individuals, society, and the economy, such as cognitive decline (1, 2). Approximately 50 million people worldwide are affected by dementia, and nearly 10 million new cases are increasing yearly, which is forecasted to triple by 2050 (3). One study predicts that the dementia population will reach 23.3 million by 2030, and the total cost of dementia is expected to reach $114.2 billion in China (4). Cognitive impairment has become a severe public health problem, placing a heavy burden on the family and society and posing significant challenges to economic development and geriatric care (5). Therefore, finding a solution to this public health issue is urgent. Growing evidence shows that caring for grandchildren is vital in exploring solutions to this problem (6–10).

With the global population aging (11), more and more older adults take on caring for grandchildren obligations (9). In a study of 10 European countries, approximately half of the grandparents provide care for their grandchildren (12). A study shows that 39% of grandparents have responsibilities for the primary caregiving of their grandchildren in the United States (13). Caring for grandchildren is also one of the necessary forms of diversified family structures and functions in China. Statistics from China Longitudinal Aging Social Survey (CLASS) 2014 suggest that 40% of older people are involved in caring for their grandchildren (14). Furthermore, some researchers proposed that providing grandchildren care positively affected cognitive function (6–10). Nevertheless, some studies also found that grandparents who provided more intensive grandchildren care showed lower cognitive scores (15, 16). Relevant studies available in Chinese people suggested that grandchildren caregiving was positively associated with cognition (7, 8). Although more attention is paid to the link between caring for grandchildren and cognitive function, the underlying mechanism still needs further exploration.

The relationship between caring for grandchildren and cognitive function may be related to social activities and depression, thus affecting cognitive function. In previous research, one study of 24 Spanish grandmothers aged 60 and over interviewers showed that, in most cases, caring for grandchildren could increase their daily activities (17). Some studies also found that caring for grandchildren could reduce grandparents' depressive symptoms (18, 19). Furthermore, numerous studies have demonstrated that older adults who are more socially engaged have higher cognitive function than those with less social engagement (20–23). In addition, some studies showed that depression was associated with an increased risk of cognitive decline (24, 25). However, the relationship and action path among depressive symptoms, social activities, caring for grandchildren, and cognition remained unknown and need further highlighting the significance of exploring the association between them.

**Figure 1:**
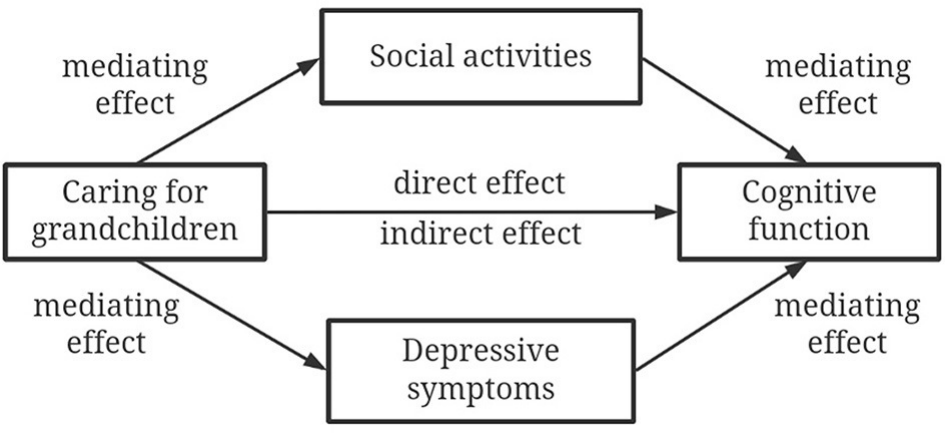
Hypothesized mediation model.

Given this, the present study aimed to (1) explore the association between caring for grandchildren based on living arrangements and cognitive function among Chinese middle-aged and older adults and (2) investigate the mediating roles of social activities and depressive symptoms in the aforementioned association. In total, one direct path and two indirect paths were hypothesized in this study (Figure 1). Particularly, caring for grandchildren would be associated with cognitive function among Chinese middle-aged and older adults. In addition, it was expected that social activities and depressive symptoms would mediate the relationship between caring for grandchildren and cognitive function.

## 2. Materials and methods

### 2.1. Study sample

Data for this study were extracted from the China Health and Retirement Longitudinal Study (CHARLS) (26), which was a nationally representative longitudinal survey hosted by Peking University. The national baseline survey for CHARLS was conducted in 2011 with 17,708 individual participants from 150 counties of 28 provinces. It has been followed up every 2 to 3 years with a Probability Proportional to Size (PPS) method, targeting residents aged 45 and above in randomly selected households and including the assessments of the social, economic, and health circumstances. The CHARLS aims to collect a set of high-quality microdata representative of Chinese households and individuals aged 45 and older to meet scientific and policy research needs on aging-related issues (26).

**Figure 2:**
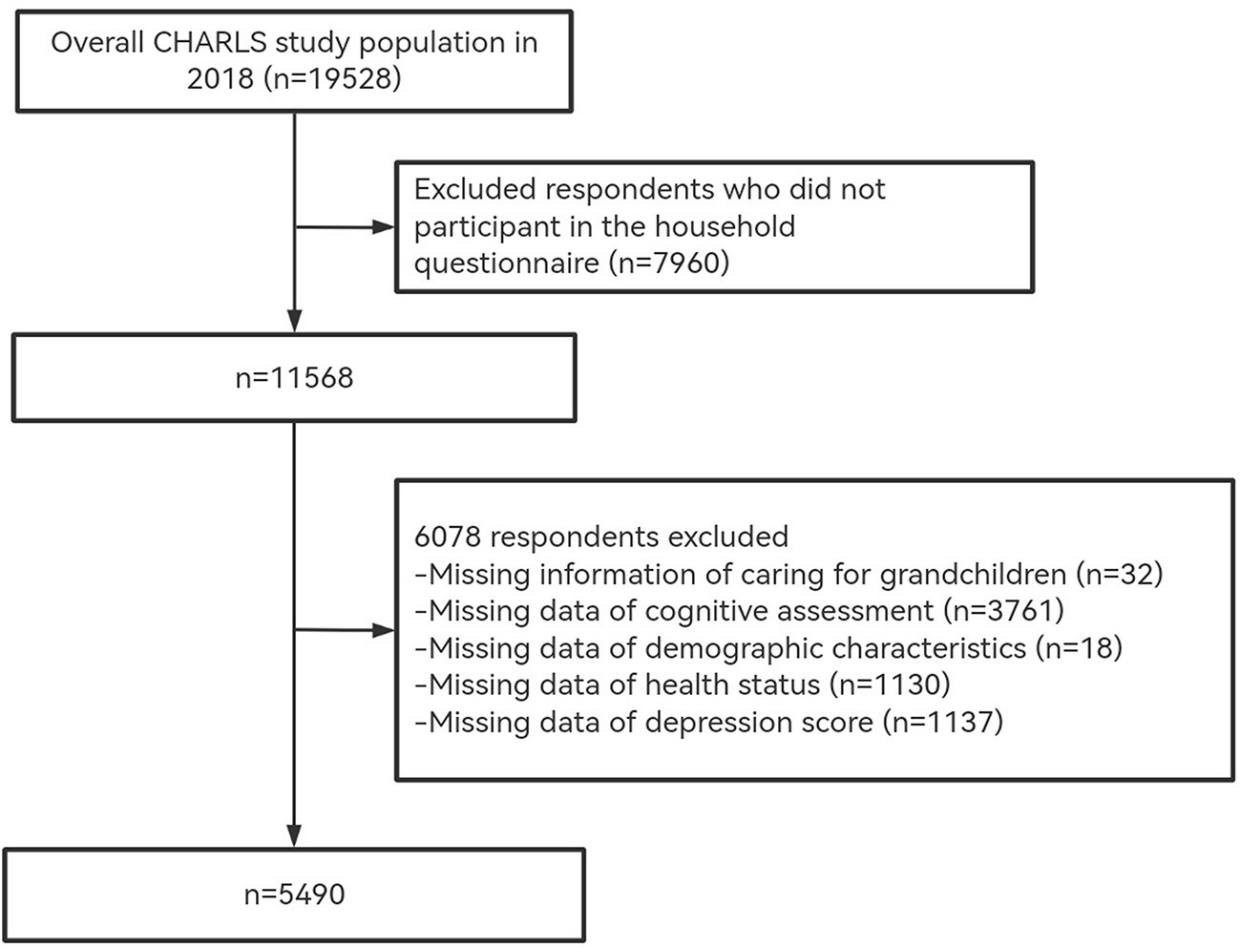
Sample selection process.

Our study used data from the CHARLS 2018 survey, including 19,528 respondents. We selected 5,490 valid participants according to the following criteria: (1) aged 45 and older; (2) provided information about caring for grandchildren; (3) completed cognitive assessments; (4) provided sociodemographic information, physical, and psychological health status. The sample selection process is shown in Figure 2.

### 2.2. Variables

#### 2.2.1. Dependent variable

The cognitive function is measured by the Chinese version of the Mini-mental State Examination (MMSE) (27). The Chinese version of MMSE has shown sufficient reliability and validity in the Chinese population and has been widely used in China for the clinical diagnosis of cognitive impairment (interrater correlation coefficient = 0.998) (28, 29). The MMSE consists of 24 items that assess orientation, episodic memory, attention and computational power, language, and visuospatial processing. The total score ranges from 0 to 30, with higher scores representing better cognitive function (30). In the CHARLS 2018, orientation is measured by time (day, month, year, season, and date of the week) and location (state, county, city or town, floor of the building, and name of the place) identification, ranging from 0 to 10 points. Episodic memory is tested using the immediate and delayed word recall methods, ranging from 0 to 6 points. The attention and computational power are assessed by asking participants to calculate 100 minus seven and keep minus seven continuously five times (0–5 points). Language skills are measured by naming objectives, repeating a sentence, following orders, reading skills, and writing a sentence (0–8 points). The visual construct is measured by redrawing a picture accurately, which had been displayed previously (0–1 point).

#### 2.2.2. Independent variable

Caring for grandchildren based on living arrangements is classified as caring for grandchildren and cohabiting with a spouse and caring for grandchildren not cohabiting with a spouse. Caring for grandchildren is measured by the following questions in the CHARLS 2018 questionnaire: (1) During last year, did you or your spouse spend time taking care of your grandchildren? It takes the value of 1 if caregiving is provided and 0 if it is not or if there are no grandchildren. (2) How many hours per week did you take care of your grandchildren? The intensity of grandchildren care is measured by categorical variables: none (0 h), non-intensive grandchild care (1–39 h), and intensive grandchild care (≥40 h) (18).

#### 2.2.3. Mediating variable

The mediating variables of this study are social activities and depressive symptoms. According to CHARLS 2018, social activities were defined as the following 11 activities in the questionnaire that respondents are involved. The frequency of participation in social events is determined by asking the respondents, “How often did you do these activities in the last month?” It takes a value of 1 if the answer is not regular; almost every week assigns a value of 2; and almost daily assigns a value of 3. Social activity intensity is measured by adding up the total score for the frequency of participation in each social activity. If not attending any social activities, it takes a value of 0. The range of frequency of social activities is from 0 to 33. Depressive symptoms are evaluated by the 10-item Center for Epidemiological Studies Depression Scale (CES-D) (31). Existing studies have shown that CES-D has good validity and reliability and CES-D can effectively measure depression levels in Chinese middle-aged and older adults (Cronbach's alpha =0.815) (32, 33). Each item uses a 4-point Likert scale. The four options are “rarely or none of the time (< 1 day),” “some or a little of the time (1–2 days),” “occasionally or a moderate amount of the time (3–4 days),” and “most or all of the time (5–7 days).” Negative symptoms are assigned as 0, 1, 2, and 3 in turn. On the contrary, two positive symptoms take values of 3, 2, 1, and 0. The scale's score is between 0 and 30, with higher scores indicating high depressive symptoms.

#### 2.2.4. Control variables

Control variables are added to this study, including age (≥45 years); gender (female = 0, male = 1); the number of types of chronic diseases was measured by whether the respondent reported having the following chronic diseases: hypertension, dyslipidemia, diabetes or high blood sugar, cancer or malignant tumor, chronic lung disease, heart problem, liver disease, kidney disease, stomach or other digestive diseases, stroke, memory-related disease, psychiatric problem, arthritis or rheumatism, and asthma (none = 0; one type of chronic disease = 1; two types of chronic disease = 2; and three types of chronic disease and above = 3); self-reported health status (very poor = 1, poor = 2, fair = 3, good = 4, and very good = 5); and life satisfaction (completely satisfied = 5, very satisfied = 4, somewhat satisfied = 3, not very satisfied = 2, and not at all satisfied = 1).

### 2.3. Data analysis

In this study, all the data analyses and processing were carried out using the IBM SPSS Statistics version 25 (Armonk, NY: IBM Corp). Mann–Whitney U-test was performed to test the differences of respondents between providing grandchildren care and non-providing grandchildren care. Multivariate linear regression analysis was performed to examine the association between caring for grandchildren based on living arrangements and cognitive function among Chinese middle-aged and older adults. Finally, the bootstrap method was used to analyze the mediation effects of social activities and depressive symptoms between caring for grandchildren and cognitive function. *P* < 0.05 was considered statistically significant.

## 3. Results

### 3.1. Basic characteristics of the respondents

Table 1 reports the basic characteristics of the whole respondents and the comparison of non-caregivers' and caregivers' subgroups. Of the entire participants, the median age of participants was 67 years old. The median score for cognitive was 21, and the median for depressive symptoms was 7. Moreover, most participants had no chronic diseases (51.6%), 51.9% of participants were somewhat satisfied with life, and 47.0% of participants reported that their health status was fair.

**Table 1:** Sample characteristics of the participants.

**Variable**	**Total**	**Caring for grandchildren**		***p***
	***n* (%)**	**No *n* (%)**	**Yes *n* (%)**	
	**5,490 (100.0%)**	**3,250 (59.2)**	**2,240 (40.8)**	
Cognitive function median (P25, P75)	21 (16, 25)	20 (15, 24)	22 (18, 26)	< 0.001
Social activity median (P25, P75)	0 (0, 3)	0 (0, 3)	1 (0, 3)	< 0.001
Gender				0.071
Female	2,850 (51.9)	1,720 (52.9)	1,130 (50.4)	
Male	2,640 (48.1)	1,530 (47.1)	1,110 (49.6)	
Age median (P25, P75)	67 (63,73)	70 (65, 76)	65 (62, 69)	< 0.001
Number of types of chronic diseases				0.735
0	2,834 (51.6)	1,666 (51.3)	1,168 (52.1)	
1 type	1,596 (29.1)	963 (29.6)	633 (28.3)	
2 types	649 (11.8)	379 (11.7)	270 (12.1)	
3 types and above	411 (7.5)	242 (7.4)	169 (7.5)	
Self-reported health status				0.014
Very poor	521 (9.5)	296 (9.1)	225 (10.0)	
Poor	616 (11.2)	353 (10.9)	263 (11.7)	
Fair	2,583 (47.0)	1,516 (46.6)	1,067 (47.6)	
Good	1,357 (24.7)	825 (25.4)	532 (23.8)	
Very good	413 (7.5)	260 (8.0)	153 (6.8)	
Depressive symptoms median (P25, P75)	7 (3, 13)	8 (4, 14)	7 (3, 13)	0.001
Life satisfaction				0.724
Completely satisfied	288 (5.2)	175 (5.4)	113 (5.0)	
Very satisfied	1,675 (30.5)	989 (30.4)	686 (30.6)	
Somewhat satisfied	2,847 (51.9)	1,667 (51.3)	1,180 (52.7)	
Not very satisfied	466 (8.5)	292 (9.0)	174 (7.8)	
Not at all satisfied	214 (3.9)	127 (3.9)	87 (3.9)	
Caring for grandchildren and cohabiting with a spouse				< 0.001
No		3,250 (100.0)	621 (27.7)	
Yes		0 (0.0%)	1,619 (72.3)	
Caring for grandchildren and not cohabiting with a spouse				< 0.001
No		3,250 (100.0)	1,619 (72.3)	
Yes		0 (0.0%)	621 (27.7)	

There were significant differences between non-caregivers and caregivers in most characteristics. The median age of those providing grandchildren care was 65 years old. Overall, 40.8% of grandparents provided grandchildren care. Among these participants, those who provided grandchildren care most cohabited with a spouse (72.3%). Moreover, the differences in cognitive function and depressive symptoms between caregivers and non-caregivers were statistically significant (*P* < 0.001). Compared with non-caregivers, caregivers scored significantly higher in cognitive function and a lower rate of depressive symptoms. Furthermore, social activities and self-reported health status significantly differed between non-caregivers and caregivers.

### 3.2. Relationship between caring for grandchildren based on living arrangements and cognitive function

As shown in Table 2, after controlling the confounding variables, caring for grandchildren and cohabiting with a spouse were positively associated with cognitive function among Chinese middle-aged and older adults (B = 0.829, *p* < 0.001). By contrast, caring for grandchildren but not cohabiting with a spouse was negatively associated with cognitive function among Chinese middle-aged and older adults (B = −0.545, *p* < 0.05).

**Table 2:** Regression results of caring for grandchildren based on living arrangements and cognitive function.

**Variable**	**Cognitive function**
	**B (95%CI)**	***p***
Caring for grandchildren based on living arrangements (reference: no care)		
Caring for grandchildren and cohabiting with a spouse	0.829 (0.498, 1.160)	< 0.001
Caring for grandchildren not cohabiting with a spouse	−0.545 (−1.001, −0.089)	0.019
R^2^ adjusted	0.213

Adjusted for age, gender, number of types of chronic diseases, depressive symptoms, self-reported health status, life satisfaction, and social activities.

### 3.3. Relationship between the intensity of caring for grandchildren based on living arrangements and cognitive function

As shown in Table 3, for those participants who cohabited with a spouse, the provision of intensive grandchildren care was more likely associated with an increase in cognitive function than those providing no grandchildren care or non-intensive grandchildren care (B = 0.843, *p* < 0.001).

**Table 3:** Regression results of the intensity of caring for grandchildren based on living arrangements and cognitive function.

**Variable**	**B (95%CI)**	***p***
Intensive of caring for grandchildren based on living arrangements (reference: no care)		
Caring for grandchildren and cohabiting with a spouse		
Non-intensive children care	0.783 (0.348, 1.219)	< 0.001
Intensive children care	0.843 (0.428, 1.258)	< 0.001
Caring for grandchildren not cohabiting with a spouse		
Non-intensive children care	−0.281 (−0.902, 0.340)	0.375
Intensive children care	−0.696 (−1.342, −0.050)	0.035
R^2^ adjusted	0.213

Adjusted for age, gender, number of types of chronic diseases, depressive symptoms, self-reported health status, life satisfaction, and social activities.

On the contrary, providing intensive grandchildren care and not cohabiting with a spouse was negatively associated with cognitive function (B = −0.696, *p* < 0.05). Nevertheless, providing non-intensive grandchildren care and not cohabiting with a spouse did not pass the statistical significance test. The regression coefficient was negative, indicating a harmful effect on the caregiver's cognitive function (B = −0.281, *p* = 0.375).

### 3.4. Mediation effect test

In Table 4, model 1 showed that the upper and lower bounds of the bootstrap 95% confidence interval of the social activities' effect did not contain 0, so it is considered that social activities played a mediating role between caring for grandchildren and cognitive function. Model 2 showed that the upper and lower bounds of the bootstrap 95% confidence interval of the depressive symptoms' effect did not contain 0, so there was also a mediating effect of depressive symptoms between caring for grandchildren and cognitive function.

**Table 4:** Table of direct effect, indirect effect, and mediation effect.

**Pathway**	**Effect**	**BootSE**	**BootLLCI**	**BootULCI**
Model 1				
Direct effect	0.500	0.153	0.200	0.800
Indirect effect	0.107	0.035	0.038	0.176
Model 2				
Direct effect	0.502	0.154	0.199	0.805
Indirect effect	0.105	0.029	0.048	0.166

**Figure 3:**
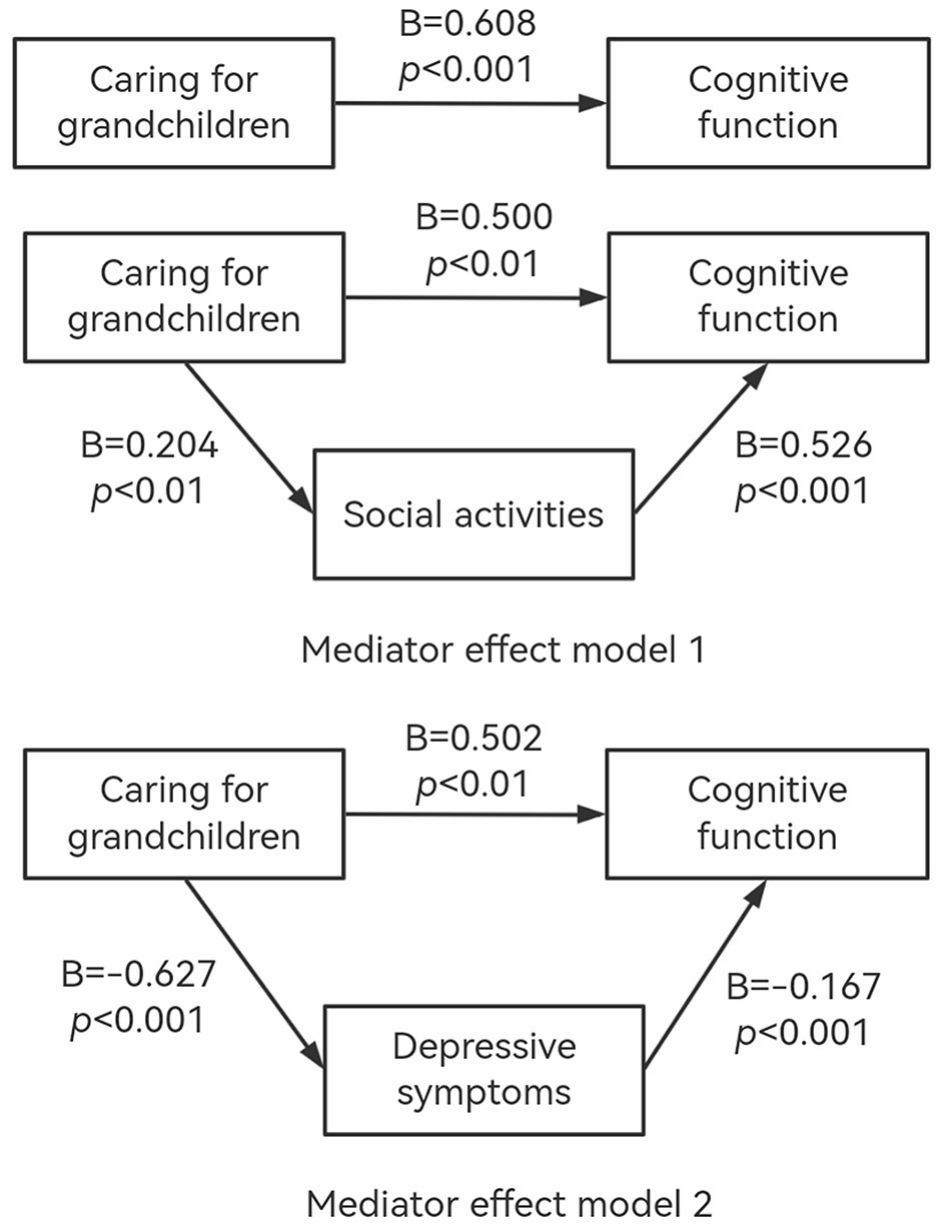
The conceptional framework of the mediation models. All models were adjusted for age, gender, number of types of chronic diseases, self-reported health status, life satisfaction.

In Figure 3, model 1 shows that there was a positive association between caring for grandchildren and cognitive function without mediating variables (B = 0.608, *p* < 0.001). After adding social activities as the mediating variable into the model, caring for grandchildren was still positively associated with cognitive function among Chinese middle-aged and older adults (B = 0.500, *p* < 0.01). In Figure 3, model 1 also reported that caring for grandchildren was positively associated with social activities (B = 0.204, *p* < 0.01). Moreover, there was a positive association between social activities and cognitive function (B = 0.526, *p* < 0.001), which meant the higher frequency of social engagement, the better cognitive function.

As shown in Figure 3, model 2, when depressive symptoms were added as a mediating variable, caring for grandchildren still had a significant positive association with cognitive function (B = 0.502, *p* < 0.01). Moreover, caring for grandchildren was negatively associated with depressive symptoms (B = −0.627, *p* < 0.001). Depressive symptoms had a significant negative association with cognitive function (B = −0.167, *p* < 0.001), which meant the lower scored depressive symptoms, the higher scored cognitive function.

Therefore, the social activities and depressive symptoms partially mediated the relationship between caring for grandchildren and cognitive function among Chinese middle-aged and older adults.

## 4. Discussion

Our findings showed that grandparents who provided intensive grandchildren care and cohabited with a spouse were more positively associated with cognitive function than those who provided no grandchildren care or non-intensive grandchildren care. Moreover, social activities and depressive symptoms partially mediated the relationship between caring for grandchildren and cognitive function among Chinese middle-aged and older adults.

This study showed that grandparents played a crucial role in China's childcare system. A total of 40.8% of Chinese middle-aged and older adults provided varying degrees of grandchildren care. The result of this study was similar to those of the studies conducted in CLASS 2014 (14) and Tang's study (34). This trend is due to China's traditional culture and the concept of filial piety. The grandparents have a moral responsibility to their children and grandchildren (35). Moreover, with the high rate of women's labor force participation in China, the conflict between work and childcare for mothers is intense (36). Therefore, Chinese parents need to rely on grandparents to help them balance work and childcare. Furthermore, in rural China, due to the surplus of agricultural labor (35), adult children often migrate to the city for better employment opportunities and leave their children with grandparents (37). In addition, there is a severe lacking of formal daycare facilities for children in China (38).

This study suggested that social activities and depressive symptoms played intermediary roles between caring for grandchildren and cognitive function among Chinese middle-aged and older adults. Our finding was consistent with some studies which showed that providing grandchildren care could reduce depressive symptoms (17, 34, 39). A cohort study showed that depression was a risk factor for dementia (40). A study by Vinkers et al. also found that cognitive impairment was associated with exacerbating depressive symptoms (24). Moreover, some studies showed that caring for grandchildren can promote mental health in older adults (34, 39). Thus, caring for grandchildren could help grandparents get emotional support, reduce depressive symptoms, and improve their cognitive function. Social activities also played a crucial role in the relationship between caring for grandchildren and cognitive function. In agreement with the previous study, our findings demonstrated a higher frequency of engagement in social interaction with a lower risk of cognitive decline (41). Based on the role reinforcement theory, caring for grandchildren as a social role provides grandparents with emotional support from grandchildren and made social connections, gaining social integration and satisfaction from social participation (42, 43). In one pilot RCT, which included social activities as an intervention component, the cognitive function of older adults was significantly improved (44). Another study found that social engagement could be viewed as a cognitively stimulating daily activity (45). Moreover, providing grandchildren care increases grandparents' opportunities for social engagement, gives grandparents more social support, and enhances their emotional health, thus promoting their cognition in social interactions (25, 34).

Caring for grandchildren and cohabiting with a spouse were positively associated with cognitive function among Chinese middle-aged and older adults. Significantly, providing intensive grandchildren care and cohabiting with a spouse were more associated with better cognitive function. Our result is consistent with the earlier studies, which showed that caring for grandchildren was positively associated with cognitive function among Chinese middle-aged and older adults (7, 8). Grandparenting is a perfect example of a social role. Cognition can be maintained due to its upbeat nature (46). Moreover, a study showed that intensive grandchildren care is associated with lower depressive symptoms (18). In addition, a study suggests that older adults without a spouse are at greater risk for depression than those with a spouse (47). The presence of a spouse can facilitate emotional communication and emotional support for older adults, thus counteracting some of the potential risks of depression (34). Empirical research proved that physiological stress and depressive symptoms are the risk factors for cognitive decline (48, 49). Therefore, by cohabiting with a spouse and caring for grandchildren, the spouse could relieve some of the physical and psychological burdens, reduce the risk of depression, and thus improve the cognitive function of the grandchildren's caregivers. In contrast, our findings suggested that caring for grandchildren but not cohabiting with a spouse was associated with a decline in cognitive function of Chinese middle-aged and older adults. A European study highlighted that intensively engaged in grandchildren care had lower cognitive function than others (16). It can be physically and psychologically demanding to help another person with daily activities (46), which may increase caregivers' stress and limit their social engagement, thus negatively affecting cognitive function (15).

The present study has certain limitations. This research uses cross-sectional data and cannot identify the causal relationship. The CHARLS questionnaire cannot determine the grandparent and grandchildren's living situation. It is impossible to analyze intergenerational caregiving's effect on caregivers' cognition in intergenerational families. Moreover, caregiver status is defined by the hours per week spent caring for grandchildren and whether the caregiver cohabited with a spouse. However, due to the database limitation, no distinction is made between co-parenting caregivers and guardians and between participating caregivers and primary caregivers. In addition, the MMSE is a self-reported screening scale, and a clinical diagnosis of cognitive impairment is unavailable. Also, there might be other potential mediators or moderators that could be further examined in future studies, for example, the parent's role in the family, the intergenerational support from children, and family cohesion.

## 5. Conclusion

This study provides evidence of the association between caring for grandchildren based on living arrangements and cognitive function among Chinese middle-aged and older adults, and it also suggests that social activities and depressive symptoms mediate the relationship between caring for grandchildren and cognitive function. The present study highlighted that caring for grandchildren and cohabiting with a spouse are positively associated with cognitive function among Chinese middle-aged and older adults. Providing intensive grandchildren care and cohabiting with a spouse are more significantly associated with better cognitive function. The findings emphasize that living arrangements, social engagement, and psychological health could be considered when encouraging grandparent care as formal care.

## Data availability statement

The datasets presented in this study can be found in online repositories. The names of the repository/repositories and accession number(s) can be found in the article/supplementary material.

## Ethics statement

The studies involving human participants were reviewed and approved by Ethical Review Committee of Peking University. The patients/participants provided their written informed consent to participate in this study. Written informed consent was obtained from the individual(s) for the publication of any potentially identifiable images or data included in this article.

## Author contributions

XH, GQ, JZ, and YX designed and conducted research. XH and YX analyzed data and wrote the manuscript. JZ and GQ revised the paper and had primary responsibility for the final content. All authors revised it critically for important intellectual content.
